# Supplementary material for: Utilising Sentinel-1’s Orbital Stability for Efficient Pre-Processing of Radiometric Terrain Corrected Gamma Nought Backscatter
Source: Sensors (Basel). 2023 Jul 1;23(13):6072. doi: 10.3390/s23136072 (PMC10346578; doi:10.3390/s23136072)
Supplement: Supplementary file 1 [file sensors-23-06072-s001.zip › sensors-2428068-supplementary.pdf]

Oversampling factor profiles

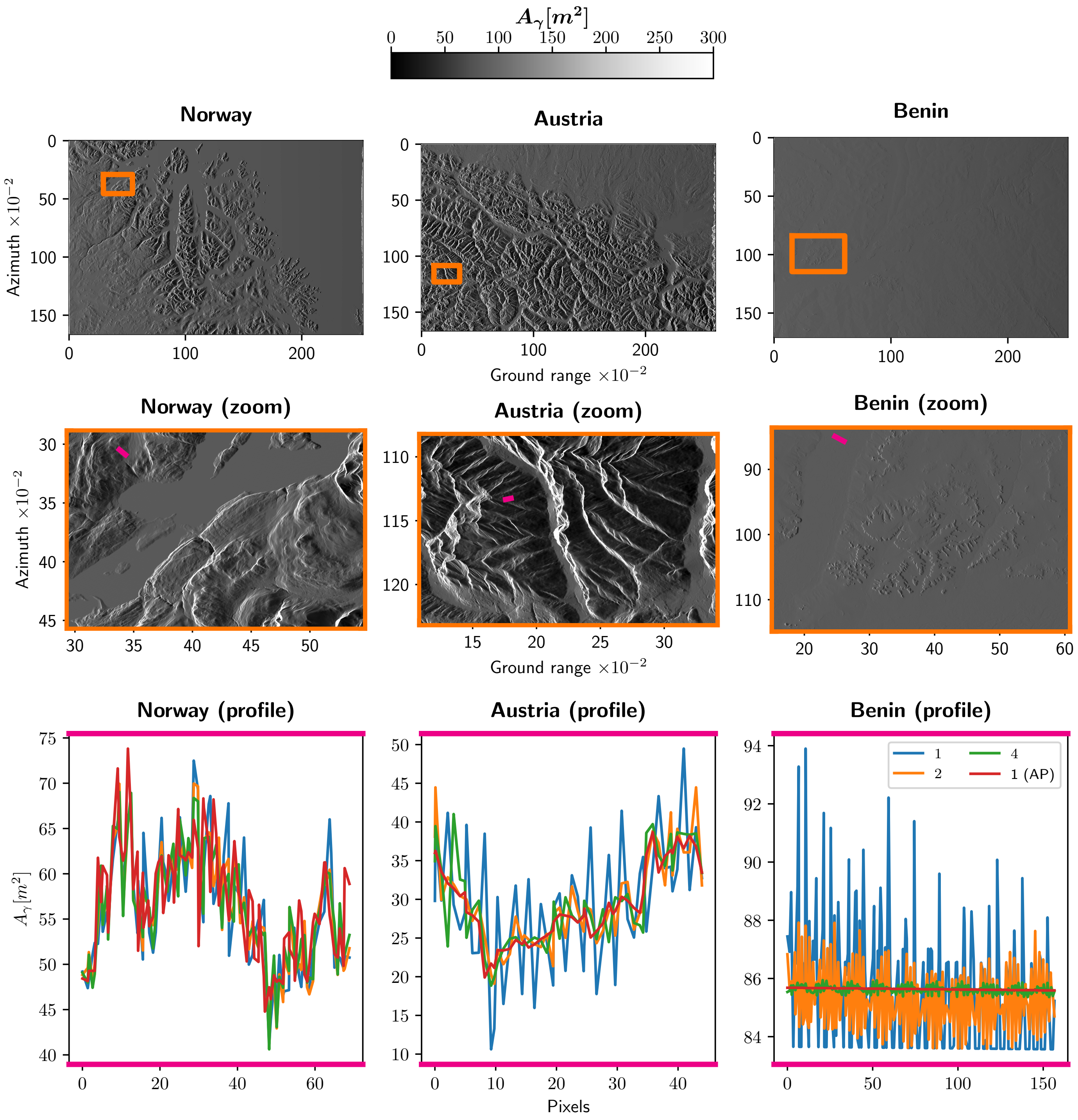

Figure 1: Demonstration how different oversampling factors are related to aliasing artefacts in the area used for radiometric terrain flattening A_γ. The first row shows A_γ in orbit geometry without oversampling and orange bounding boxes delineating the zoom-in view of the second row. The last row displays A_γ values along the pink profile drawn in the center row for the oversampling factors 1, 2, 4, and 1 with Area Projection (AP).

According to the analysis of the oversampling factor in our main manuscript and under the given observation geometry and terrain conditions, an oversampling of at least 4 is needed to fulfill the Nyquist–Shannon sampling theorem. Figure 1 demonstrates the impact of certain oversampling factors below the minimum required value of 4. The lower the oversampling factor, the more dominant, jumpy artefacts are visible along the A_γ profile. These artefacts propagate directly into the final γ_T^0 product, significantly reducing its radiometric quality, and disappear when high oversampling factors are applied at a cost of run time performance. Fortunately, with the novel Area Projection (AP) algorithm it is possible to visually outperform the other A_γ profiles and thus eliminate the need for oversampling.
